# The pristine unused pulmonary surfactant isolated from human amniotic fluid forms highly condensed interfacial films

**DOI:** 10.14814/phy2.70403

**Published:** 2025-06-19

**Authors:** Juan Carlos Castillo‐Sánchez, Ainhoa Collada, Emma Batllori‐Badia, Alberto Galindo, Antonio Cruz, Jesús Pérez‐Gil

**Affiliations:** ^1^ Biochemistry and Molecular Biology Department, Faculty of Biology Complutense University Madrid Spain; ^2^ Research Institute Hospital 12 de Octubre (Imas12) Madrid Spain; ^3^ Department of Public and Maternal‐Child Health, Faculty of Medicine Complutense University of Madrid, 12 de Octubre University Hospital Madrid Spain; ^4^ Maternal and Child Health and Development Research Network (RICORS‐SAMID Network) Ref. RD21/0012/0024 Madrid Spain

**Keywords:** air–liquid interface, epifluorescence microscopy, lipid phases, lung surfactant, monolayers, surface tension

## Abstract

Pulmonary surfactant is a lipid/protein complex that coats the alveolar air–liquid interface to minimize surface tension facilitating breathing mechanics. Native surfactant (NS) is typically obtained from lavages of animal lungs, where it has gone through structural alterations as a result of exposure to respiratory dynamics and highly oxidative environments. We have studied here the structure of interfacial films formed by human amniotic fluid surfactant (AFS), thought to maintain the structural and functional features of a fully operative still non‐used surfactant, as it has not been subjected to breathing dynamics yet. The results show that AFS adsorbs better at the interface, to form films supporting higher compression rates, than NS upon spreading at comparable concentrations and amounts. Films formed by AFS exhibit condensed regions excluding fluorescently labeled lipids from the mere adsorption, while films formed by NS only showed segregation of ordered‐like domains once subjected to compression–expansion dynamics. Finally, AFS films were consistent with the presence of solid‐like highly ordered phases, while NS consisted under comparable conditions of a coexistence of liquid‐disordered/liquid‐ordered fluid phases. This indicates that operative surfactant films formed by freshly secreted surfactant could be much more condensed than previously supposed, likely providing maximal stability under breathing mechanics.

## 1 INTRODUCTION

The surface tension (*γ*) at the respiratory air–liquid interface is minimized by a lipid/protein complex named pulmonary surfactant (PS). In general terms, PS consists of an adsorbed monolayer connected to a membrane reservoir able to provide the interface with newly synthesized complexes or lipids previously excluded from the interface when the respiratory surface increases (during inspiration). These structures and their reorganizations are possible thanks to PS composition: 90% (w) lipids, mainly phospholipids, and around 10% (w) of some surfactant‐associated proteins. Hydrophobic surfactant proteins SP‐B and SP‐C play key roles in the formation and dynamics of these surface‐associated reservoirs by mediating lipid/protein and lipid/lipid interactions and facilitating lipid trafficking toward and from the interface (Lopez‐Rodriguez & Pérez‐Gil, 2014).

A reduction of *γ* to minimal values (around 1 mN/m) at the end of exhalation (when the respiratory surface decreases) is necessary for maintaining air patency and sustaining operational breathing. To reach minimal *γ*, the lateral structure of interfacial PS monolayers evolves from disordered‐like lipid phases at equilibrium to a highly ordered interfacial film with maximal packing (and minimal exposure of water to air) at minimal surface area. To achieve that, it is believed that condensed domains start forming as nucleation points that grow upon compression. This occurs for model monolayers constituted by pure lipids such as dipalmitoylphosphatidylcholine (DPPC), the major and most surface‐active surfactant component, which segregates, upon compression, liquid‐condensed (*L*_c_) and liquid‐expanded (*L*_e_) lipid phases. Furthermore, cholesterol‐containing materials, as is the case of PS, organize in a liquid‐ordered (*L*_o_)/liquid‐disordered (*L*_d_) type of coexistence, with a fluid character and differential interfacial performance (Baoukina et al., 2012): *L*_o_ domains can adopt spontaneous curvature at low *γ* favoring monolayer collapse (Baoukina et al., 2012, 2014). Therefore, composition (specifically cholesterol and DPPC content) and compression are driving factors modulating lipid‐phase segregation in interfacial surfactant films (Baoukina et al., 2012) and eventually the squeeze out of the less stable lipids during compression that is necessary to attain the minimum *γ* required for breathing (Cruz et al., 2004; Zhang et al., 2011).

Not only lipids influence lateral phase segregation but also surfactant hydrophobic proteins play specific roles in this regard. SP‐B seems to modify the distribution of condensed domains, maximizing *L*_c_/*L*_e_ boundaries (Cruz et al., 1997, 2000, 2004; Krol et al., 2000; Nag et al., 1997; Takamoto et al., 2001) and stabilizing compression‐induced 3‐D membranous reservoirs (Cruz et al., 1997; Krol et al., 2000; Lee et al., 2001; Nag, Perez‐Gil, Cruz, Rich, & Keough, 1996; Taneva & Keough, 1994a; Worthman et al., 2000). Similarly, protein SP‐C also partitions into disordered‐like lipid phases, decreasing condensed domain size while prompting multilayer growth near collapse (Kramer et al., 2000; Na Nakorn et al., 2007; Nag et al., 1997; Nag, Perez‐Gil, Cruz, & Keough, 1996; Nag, Perez‐Gil, Cruz, Rich, & Keough, 1996; Plasencia et al., 2005; Takamoto et al., 2001; von Nahmen et al., 1997; Wang et al., 2005). Furthermore, the hydrophilic protein SP‐A expands interfacial PS films forming a condensed network along with SP‐B (Ridsdale et al., 2001; Taneva & Keough, 2000; Worthman et al., 2000; Zuo, Keating, et al., 2008) and facilitates 2D‐to‐3D conversion on compression (Zuo, Tadayyon, et al., 2008).

The particular properties and effects of the different components in PS have been explored over the years, typically through the analysis of relatively simplified lipid and lipid/protein models. However, the sophisticated nature of PS performance at the respiratory surface can only be apprehended through the study of the fully complex native materials. Natural PS complexes thus give rise to interfacial films whose complexity is greater than those formed by model mixtures (Discher et al., 1999; Nag et al., 1998; Sachan & Zasadzinski, 2018). When PS is assembled and secreted as lamellar bodies (LBs) by primary cultures of type II cells, the *lamellar body‐like secreted particles* (LBPs) form films with highly complex three‐dimensional structures and a solid‐like character (Cerrada et al., 2015). In contrast, PS preparations obtained from lavage also adsorb rapidly into air–liquid interfaces but form much simpler flat films (Ravasio et al., 2010). Until a few years ago, the limited amount of material available from cell cultures had prevented the extensive analysis of the structure and quality of the films formed by freshly secreted surfactant. The recent discovery that unused PS complexes could be purified in relatively high yields from human amniotic fluid (Castillo‐Sánchez, Roldán, et al., 2022) opened new opportunities to explore key structure‐functional determinants of a truly operative surfactant. That amniotic fluid surfactant (AFS) was characterized extensively in a recent work, presenting fully comparable composition to that of surfactant isolated from lavaged lungs (NS) (see a summary of comparative compositional data in Table 1). Particularly, it is remarkable that both surfactants have similar proportions of phosphatidylcholine (PC) (86% in NS, 89% in AFS) and its main disaturated species DPPC (33% in NS, 34% in AFS), as well as comparable proportions of saturated/unsaturated and zwitterionic/anionic phospholipid species.

**TABLE 1:** Comparative lipid composition of NS and AFS.

	NS	AFS
Phospholipid class (mol%)		
PC	86.4 ± 0.7	89.1 ± 0.6
DPPC	32.6 ± 0.8	34.4 ± 0.8
PG	1.6 ± 0.2	2.1 ± 0.4
PE	6.3 ± 0.6	4.0 ± 0.4
PI	4.7 ± 0.4	2.6 ± 0.7
Cholesterol (w/w vs. PL)	4.0 ± 1.0	9.9 ± 1.9

*Note*: Phospholipid composition of NS and AFS (*n* = 3) in molar proportion as determined by HPLC and mass spectrometry. Cholesterol content is expressed with respect to phospholipid mass. Elaborated with data from Castillo‐Sánchez, Roldán, et al. (2022).

Interestingly, phosphatidylinositol (PI) is the dominant anionic species in the porcine surfactant whereas phosphatidylglycerol (PG) is the main negatively charged phospholipid in the human material. It is also remarkable that human surfactant contains more cholesterol (around 10% with respect to phospholipid mass) than porcine surfactant (3%–5%). However, in spite of that similar composition, AFS is characterized by a much more complex, multilayered, and dehydrated structure, melting at remarkably higher temperatures (Castillo‐Sánchez, Roldán, et al., 2022). With such a structure, AFS was reported to exhibit enhanced interfacial properties compared with NS, in terms of adsorption kinetics and of maintenance of very low surface tension under breathing‐like compression–expansion dynamics.

With this in mind, the goal of the current study has been the detailed characterization of the surface activity and the lateral organization and three‐dimensional transitions of interfacial films formed by human AFS, in comparison with the films typically formed by NS. The results we describe here confirm that pristine unused surfactant such as AFS preserves an intrinsic ability to adsorb very efficiently into the interface, forming films that are much more condensed, packed, and prone to form three‐dimensionally complex solid layers than surfactant obtained from lavaged lungs, which has been presumably already “spent” as a consequence of breathing dynamics and air exposure.

## 2 MATERIALS AND METHODS

### 2.1 Surfactant complexes

Pulmonary surfactant complexes were purified from different natural sources following protocols optimized in our laboratories (Taeusch et al., 2005). For NS, porcine lungs obtained at the slaughterhouse from pigs destined for human consumption (with a variety of age, sex and conditions) were washed with a buffer solution Tris 5 mM, NaCl 150 mM pH 7 to obtain the bronchoalveolar lavages. Cellular debris was removed by a short centrifugation (1000× *g*, 5 min, 4°C). Then, supernatants were centrifuged at 100,000× *g*, 4°C for 1 h to obtain surfactant complexes in the pellets, that were homogenized using a potter and charged into a sodium bromide discontinuous density gradient containing three solutions: from bottom to the top, 4 mL of NaBr 16% (w/v) in NaCl 0.9%; 6 mL of NaBr 13% in NaCl 0.9%; and 2.5 mL NaCl 0.9%. Density gradients were centrifuged at 120,000× *g*, 4°C for 2 h, and surfactant complexes were harvested from the interface between the second and third gradient solutions and diluted in NaCl 0.9%.

To purify AFS, amniotic fluid was obtained from full‐term programmed cesareans in collaboration with the Obstetrics and Gynecology Service of Hospital 12 Octubre (Madrid, Spain), as previously explained (Castillo‐Sánchez, Roldán, et al., 2022). Written informed consent was presented and approved by the Ethical Committee of Hospital 12 Octubre (CEI 16/205—14/07/2016). Inclusion criteria considered normal pregnancy outcomes, undergoing scheduled cesarean section at term for obstetric reasons. Donor mothers included in the study presented a mean age of 33.1 ± 5.1 yr. Any maternal or fetal complication during pregnancy leads to exclusion from the study. AFS was purified from the human amniotic fluid following the same protocol described above for the purification of NS.

### 2.2 Langmuir balance and epifluorescence microscopy

Aqueous suspensions of PS were labeled with the fluorescent probes NBD‐PC (Cat. NC1108215, ThermoFisher Scientific), 1% mol/mol with respect to phospholipid and/or DiIC18 (Cat. D12731, 0.5% molar) for 1 h at 45°C with orbital agitation. Then, enough PS was deposited on the interface of a Langmuir surface balance 302RB/D1 (Nima Technology) equipped with a thermostated trough of 213 cm^2^, containing 400 mL of buffer 150 mM NaCl 5 mM Tris, pH 7.4, to reach detectable Π values of 1–2 mN/m. Films were then equilibrated for 10 min, and isotherms were acquired at 25°C, at 25 cm^2^/min, before or after five compression–expansion cycles run at 65 cm^2^/min. The spreading of materials directly as aqueous suspensions allowed preserving their original structure while comparing their efficacy to transfer surface‐active components into the air–liquid interface. In our hands, the careful spreading of the best surfactant preparations, such as NS or AFS, as aqueous suspensions at the surface of the trough, may end in their almost quantitative transfer and association with the interface.

For observation of the films by epifluorescence microscopy, interfacial films were transferred onto de‐lipidized glass coverslips while continuously varying Π to obtain the so‐called COVASP (continuously varying surface pressure) supported layers, as previously described (Wang et al., 2007). This methodology has been shown to preserve, in a long‐term immobilized form, the lateral structure of films compressed to any surface pressure along the isotherm, under conditions that allow the acquisition of high‐resolution fluorescence images from several coexisting probes. Fluorescence images were acquired with an ORCA R2 10600 camera (Hamamatsu Photonics K.K.) coupled to an epifluorescence microscope Leica DM 4000B (Leica Microsystems) to scan the whole range of Π equipped with L5 (ranges of excitation and emission 440–520 nm and 497–557 nm, respectively) and TX2 (520–600 nm and 670–720 nm excitation and emission ranges) Leica filter sets. Images were taken at 40× (Leica Germany) using the specific software LASAF (Leica Application Suites, Leica Microsystems). Sampling, exposure, and normalization according to histograms were all made under comparable conditions for all the images to facilitate their comparison.

Different batches of NS and AFS were used to perform the experiments, and several images were taken for each surface pressure in the isotherms of each of the batches. In consequence, representative images have been used to compose the figures. Isotherms and data from images were never averaged between different biological replicates because they were never obtained exactly at the same pressures. However, the structure and behavior of films from different NS and AFS samples resulted to be highly comparable. Phospholipid quantification in each batch was performed by phosphorus quantification following the protocol described by Rouser et al. (1966). ImageJ was used for quantitative measurements of the number, areas, and perimeters of segregated domains within the transferred films. Data presented in the figures are given as an orientation of the magnitude of the number and size of the domains observed along the isotherm, with no statistical meaning because they are not obtained from averaging truly biological replicates. Numbers were actually the average of measurements repeated three times in each of five different frames at each indicated pressure.

## 3 RESULTS

**FIGURE 1:**
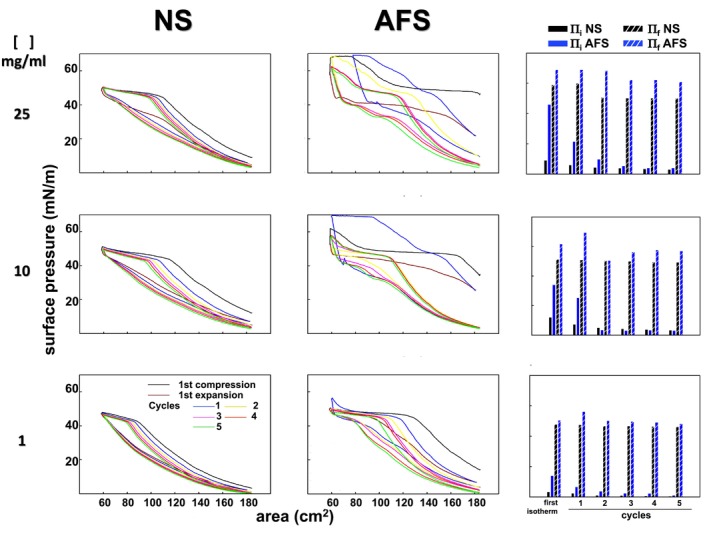
Cyclic Π‐area isotherms from interfacial films formed by NS and AFS. Samples of NS (left) or AFS (center) were spread at the interface of the surface balance at a concentration of 25 (top), 10 (middle), and 1 mg/mL (bottom). Total phospholipid amount injected was in all cases 50 μg. The acquisition of a first compression Π‐area isotherm was recorded at 25 cm^2^/min, as well as the subsequent full area expansion and surface cycling (5 compression–expansion cycles at 65 cm^2^/min). Right) Initial and final Π for each compression Π‐area isotherm and cycle.

Cyclic compression–expansion Π‐area experiments were performed upon spreading of three different concentrations of the surfactants, 25, 10, or 1 mg/mL, in order to evaluate the full range of Π (Figure 1). As expected considering the higher surface activity of AFS (Castillo‐Sánchez, Roldán, et al., 2022), the initial Π, 10 min after deposition, was higher for AFS than for NS at all the concentrations used: in the example illustrated in Figure 1, 46 versus 9 mN/m at 25 mg/mL, 34 versus 12 mN/m at 10 mg/mL, and 14 versus 3.4 mN/m at 1 mg/mL. Consequently, for surfactant suspensions spread at 10 or 25 mg/mL, AFS films reached Π above the exclusion plateau (45 mN/m) during the first compression, whereas maximal Π for NS was below 48 mN/m for all the phospholipid concentrations tested. Despite higher initial Π, AFS suspensions spread at 1 mg/mL allowed the acquisition of full compression Π‐area isotherms all the way from low Π to the exclusion plateau. We therefore set this phospholipid concentration to extensively compare NS and AFS films in the subsequent experiments. Moreover, cycling compression Π‐area isotherms were qualitatively comparable for NS and AFS. Overall, note that, as it could be expected and despite depositing the same amount of phospholipid (50 μg, total mass), the lower the concentration spread, the lower the Π reached.

**FIGURE 2:**
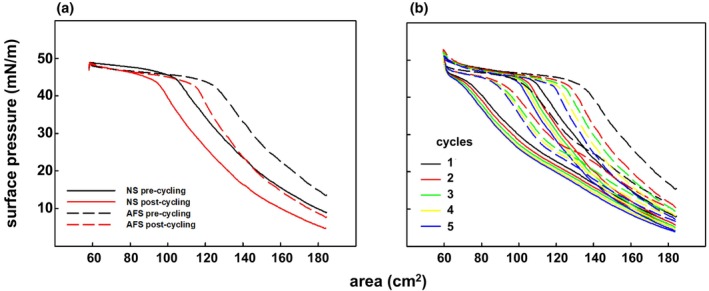
Compression–expansion Π‐area isotherms of interfacial films formed by NS and AFS. (a) Compression isotherm of NS (solid lines) and AFS (dashed lines) labeled with the fluorescent probe NBD‐PC (1%, molar) and acquired at 25 cm^2^/min before (black) and after (red) compression–expansion cycling. 50 μg (total phospholipid mass) was injected at the surface of the trough at 1 mg/mL. (b) Cyclic Π‐area isotherms for interfacial films of NS (solid lines) and AFS (dashed lines) acquired upon compression–expansion cycling at 65 cm^2^/min. Five consecutive cycles were performed: first (black), second (red), third (green), fourth (yellow), and fifth cycle (blue).

**FIGURE 3:**
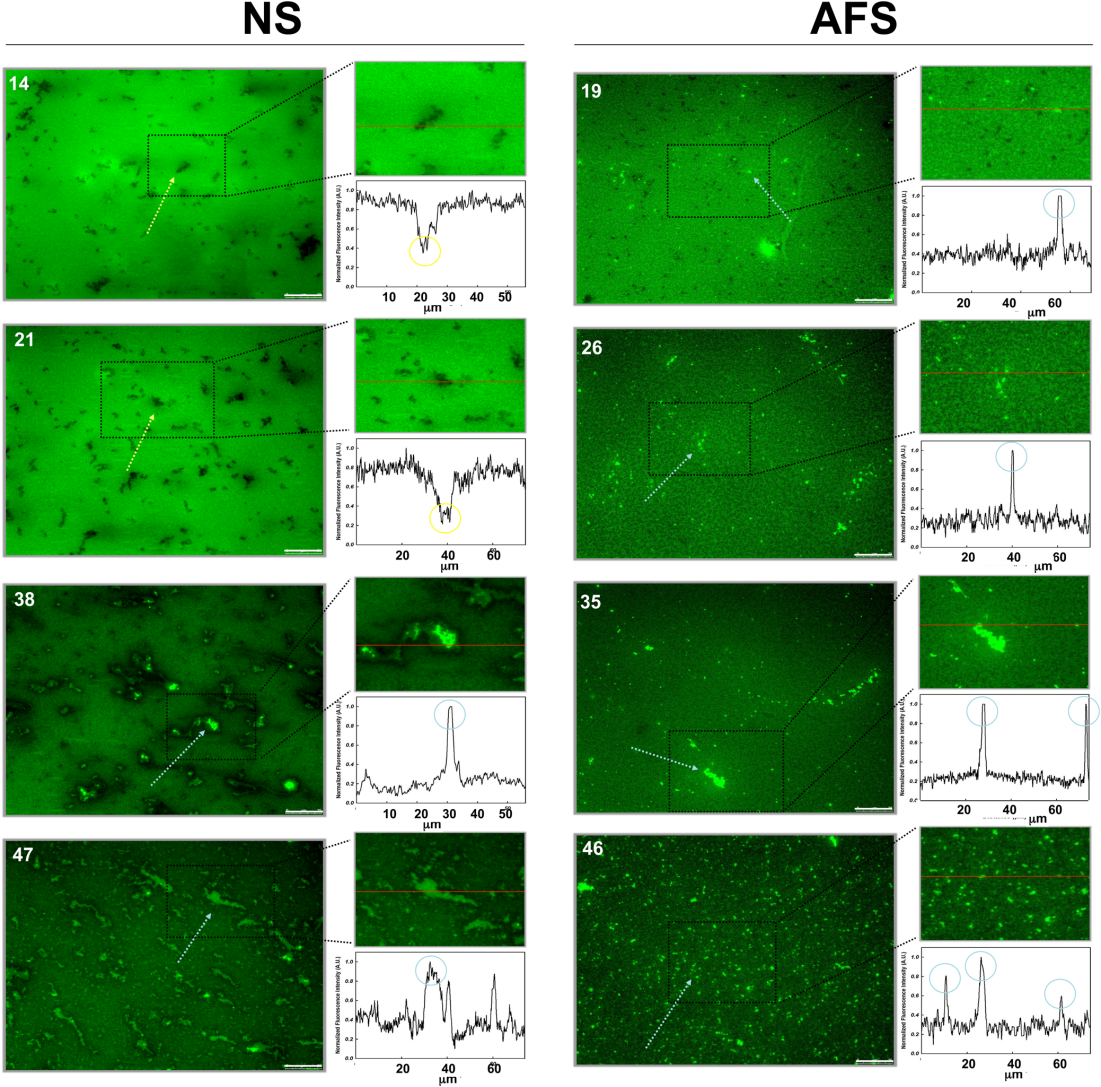
Epifluorescence images of interfacial films made by NS and AFS before surface cycling. Epifluorescence images were taken at the pressures indicated (in mN/m) from films transferred by the COVASP method before surface cycling for NS (left) and AFS (right). Normalized fluorescence profiles at the right lower side of each image correspond to the red line drawn in the region amplified above of each profile. Label‐free stringy regions and label‐enriched exclusion spots are indicated with yellow and blue arrows, respectively (within the fluorescence images), and circles (in the normalized fluorescence profiles). For clarity, from a minimum of three images obtained per surface pressure, only one representative image per pressure is shown. Scale bars mean 25 μm.

**FIGURE 4:**
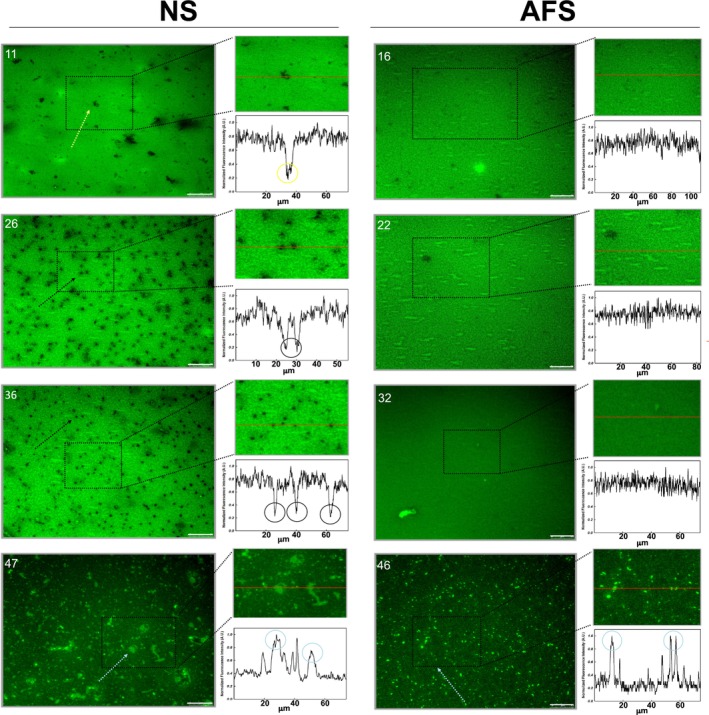
Epifluorescence images of interfacial films of NS and AFS after surface cycling. Epifluorescence images were taken at the pressures indicated (in mN/m) from films transferred by the COVASP method after five compression–expansion cycles for NS (left) and AFS (right). Normalized fluorescence profiles at the right lower side of each image correspond to the red line drawn in the region amplified above of each profile. Label‐free stringy regions, label‐free condensed domains, and label‐enriched regions are indicated with yellow, black, and blue arrows (within the fluorescence images) and circles (in the normalized fluorescence profiles), respectively. For clarity, from a minimum of three images obtained per surface pressure, only one representative image per pressure is shown. Scale bars mean 25 μm.

Then, we performed experiments using surfactant complexes labeled with the fluorescent probe NBD‐PC (1%, molar) (Figure 2). Compression Π‐area isotherms, obtained before and after surface cycling, were comparable to those obtained from non‐stained AFS or NS, with AFS always exhibiting higher Π than NS. During the compression process, the interfacial films were simultaneously transferred onto glass coverslips following the COVASP method (Wang et al., 2007), while varying and registering Π continuously, for imaging the transferred films compressed to any stage of the isotherm under an epifluorescence microscope (Figures 3 and 4).

**FIGURE 5:**
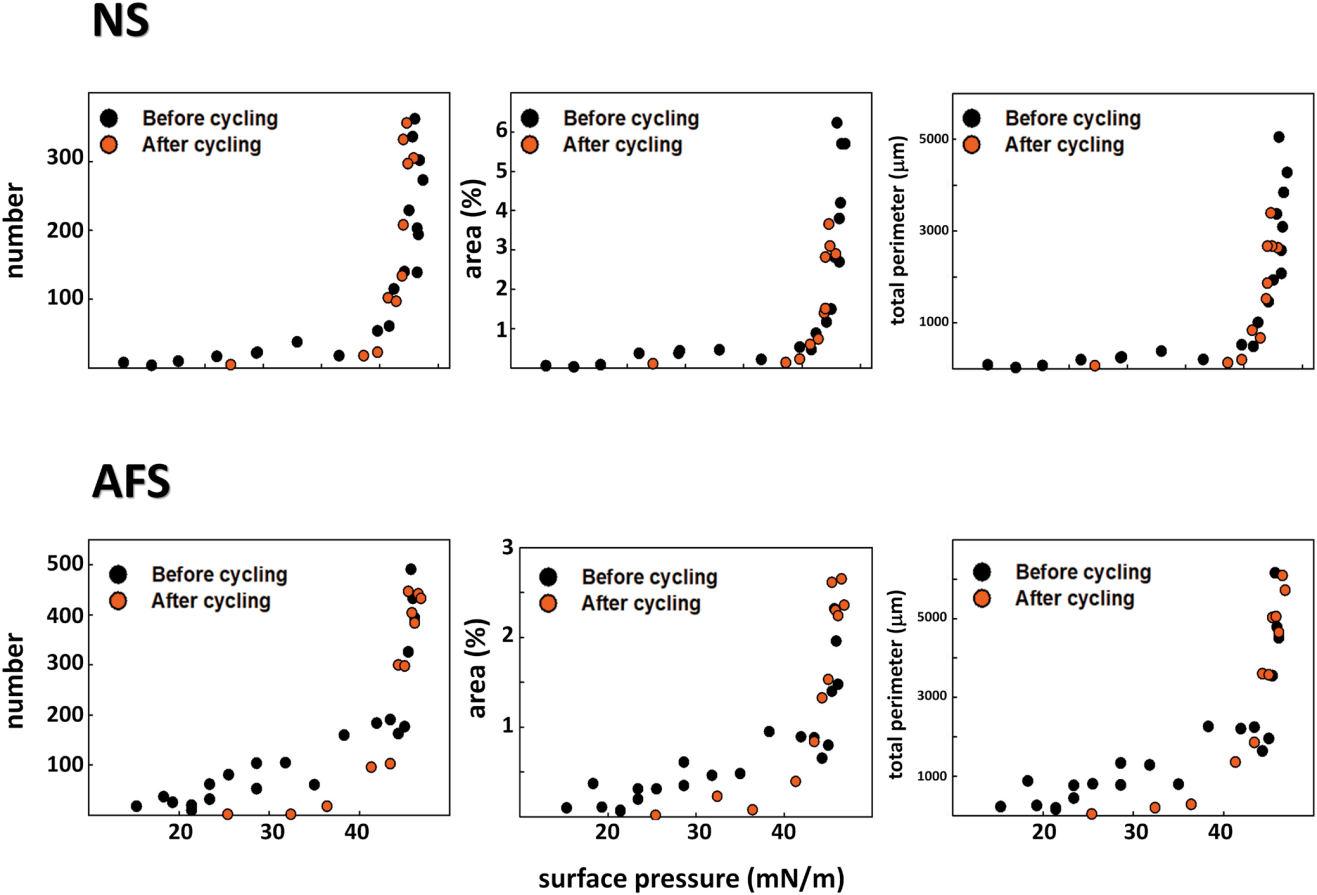
Quantitative analysis of label‐enriched regions segregated in interfacial films of NS (top) and AFS (bottom). Number (left), total area percentage (center), and total perimeter (right) of label‐enriched spots (marked by blue arrows in Figures 3 and 4) are plotted as a function of Π before (black) and after (orange) compression–expansion cycling. Values plotted in the figures represent the mean value for three different counts of five frames per pressure.

On the one hand, prior to surface cycling, interfacial NS films formed a disordered‐like lipid phase at low Π where NBD‐PC appears homogenously distributed within the film (Figure 3, left panels). Moreover, filamentous worm‐like label‐free regions were observed in NS films compressed to pressures between 10 and 21 mN/m (yellow arrows in Figure 3). Specifically, we observed in the order of 100 of these label‐free regions per fluorescence image, accounting for 2%–3% of the total area. At pressures above 35 mN/m, bright spots started to be observed (blue arrows in Figure 3), apparently accumulating fluorescent probe. Indeed, the number of these label‐enriched spots per frame increased dramatically just below the plateau, for example, 360 fluorescent spots accounting for about 7% of the total area and 5000 μm of perimeter at 48 mN/m (quantitated in Figure 5). These regions likely represent compression‐driven 3‐D lipid protrusions containing unsaturated lipids and producing high light scattering, since they are formed during the isotherm plateau, which likely corresponds to the squeeze‐out of lipids and proteins (Taneva & Keough, 1994b, 1994c, 1994d). Furthermore, NBD‐PC, which partitions into disordered‐like lipid phases, seems to accumulate there, probably associated with a more disordered state of lipids in these protrusions than occurring at the DPPC‐enriched interfacial regions in a more ordered‐like lipid phase, which is likely excluding a fair amount of probe, and becomes thus darker.

**FIGURE 6:**
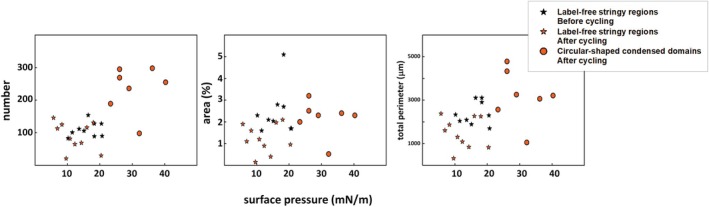
Quantitative analysis of label‐free regions segregated in interfacial films of NS. Number (left), total area percentage (center), and total perimeter (right) of label‐free segregated regions (marked by yellow and black arrows in Figures 3 and 4) are plotted as a function of Π before (black) and after (orange) compression–expansion cycling. Black stars represent label‐free stringy regions before surface cycling, orange stars after surface cycling and orange circles refer to circular‐shaped condensed domains only formed after surface cycling. Values plotted in the figure represent the mean value for three different counts of five frames per pressure.

After five compression–expansion cycles, the interfacial NS films displayed a different structural pattern. Apart from the absence now of the worm‐like label‐free regions at low Π, we observed circular‐shaped condensed domains appearing at Π ranging from 23 to 40 mN/m (black arrows in the left panels of Figure 4 and orange circles in Figure 6). Indeed, the number of such condensed domains was higher than that of the filamentous label‐free regions: 80–150 versus 100–300 (Figure 6). This condensed lipid phase accounted for 2%–3% of the interfacial area with a total perimeter in the order of 2500–5000 μm (Figure 6). Moreover, the disordered‐like phase was structured as a network‐like (look at images at 26 and 36 mN/m), suggesting extensive ordered/disordered‐like lipid phase coexistence. However, without being obvious, it cannot be excluded that the interfacial NS film was already organized to sustain lipid coexistence before surface cycling. Lastly, label‐enriched regions were similarly observed on compression after surface cycling, even though they appeared at higher Π than observed in films before being subjected to surface cycling: 43 versus 23 mN/m (Figure 5). Despite their similar number, the area percentage occupied by these label‐enriched regions and the total perimeter were lower than observed in NS films before cycling: up to 3.6% versus 6.2% and 3400 versus 5000 μm, respectively (Figure 5).

On the other hand, AFS apparently produced much more elaborated interfacial films just after spreading and adsorption or once cycled (right panels in Figures 3 and 4). Both before and after surface cycling, we observed that AFS forms particularly complex and reticular interfacial films comprising probably at least two well‐segregated lipid phases (see images at 19 and 26 mN/m, for instance). Furthermore, we observed bright label‐enriched regions not only just below the isotherm plateau but also at low Π (between 15 and 30 mN/m) and prior to surface cycling but not after (Figures 3 and 4). Specifically, we observed around 100 circular‐shaped, small, label‐enriched spots at Π in the range 15–35 mN/m (Figure 5). These label‐enriched spots accounted for up to 0.5% (area percentage), defining a total perimeter of 400 μm maximum (Figure 5).

As for NS films, there was a dramatic increase of those label‐enriched regions at Π corresponding to the compression Π‐area plateau, that is, from 40 mN/m onwards, before and after surface cycling (reaching a maximum of 500 bright regions) (Figure 5). Conversely, their dimensions were similar before and after cycling, accounting for up to 2.5% of surface area and 3000 μm of total perimeter. Compared to NS films, the exclusion regions in AFS were more numerous (up to 500 vs. 400), smaller, circular‐shaped, and occupying less fraction of the surface area (up to 2.5% vs. 6%). Still further, and in contrast to NS, we did not observe circular‐shaped condensed domains in interfacial AFS films after surface cycling.

**FIGURE 7:**
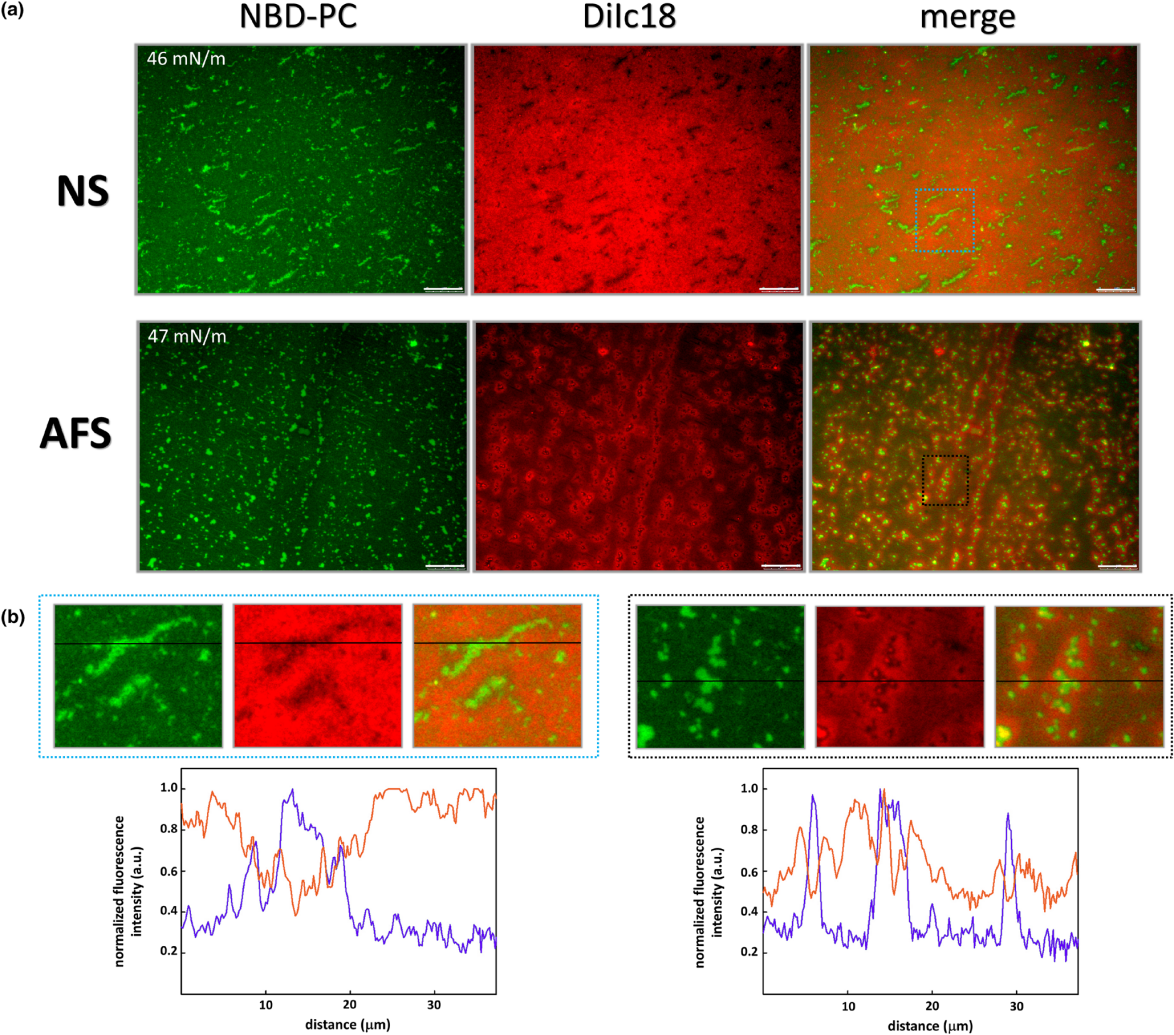
Epifluorescence images of interfacial films of NS and AFS labeled with NBD‐PC and DiIC18. (a) Representative images were recorded at the indicated pressures from transferred COVASP films observed independently under the NBD‐PC (left, green fluorescence) and the DiIC18 (middle panel, red fluorescence) channels, which have been merged in the right frames. (b) Zoom of regions indicated by dotted squares in the fluorescence images of NS (left) and AFS (right) films. Normalized fluorescence profiles correspond to the black solid lines drawn in the images. Scale bars mean 25 μm.

To further understand the differences observed between NS and AFS, we conducted experiments simultaneously including NBD‐PC and DiIC18 probes that partition into fluid disordered and ordered‐like lipid phases, respectively. From these experiments, our results suggest that the compression‐driven processes occurring in NS and AFS films are remarkably different (Figure 7). On the one hand, in NS films, the fluorescence signal of DiIC18 seems to be distributed along the whole surface area except for regions where NBD‐PC was accumulated. This feature suggests that, at around 45 mN/m, unsaturated lipids could be accumulated and somehow excluded (NBD‐PC fluorescence) whereas the surface area was structured as a relatively homogeneous ordered‐like lipid phase (DiIC18 signal). This means that films of NS could still maintain, at high compression, a relatively fluid character differentiating a base of fluid ordered phase and regions where probe‐rich disordered phase could be prepared to initiate 2D to 3D transitions upon further compression. In contrast, Figure 7 illustrates how compression of AFS films seems to cause the partition of both fluorescent dyes into specific regions, which seem to show an NBD‐PC‐enriched core surrounded by a DiIC18‐labeled area. This suggests that the squeeze‐out pattern of fluid lipids is much more elaborated in AFS films, including segregation of disordered‐like regions enclosed by ordered‐like lipid phases to let a practically label‐free area covering the rest of the interfacial film. As a consequence, AFS films seem to reach more condensed solid‐like states, which exclude all the probes, and presumably produce the highest Π, that is, the lowest *γ*.

The intrinsic difference in complexity and level of condensation between films formed by AFS and NS could be well appreciated in all the different batches analyzed of the two types of materials (see Figure S1, where images at two different surface pressures are summarized for two other different batches of either NS or AFS, simultaneously labeled with NBD‐PC and DiC18). Analogously to the differences in structure described in Figures 3, 4, 5, 6, 7, it is clear in Figure S1 how films formed by AFS exhibit a higher complexity, with more different phases in terms of levels of segregation of the two incorporated probes, and substantially higher levels of condensation/packing than observed in NS films. This is evident from the presence in AFS films of regions excluding completely the two probes, which were never observed in NS films, and the much more numerous bright spots corresponding to scattered/squeezed out 3D aggregates excluded from the interface at a relatively moderated surface pressure of 30 mN/m.

Despite being an easy and useful methodology, it is important to note that the imaging of interfacial PS films under epifluorescence microscopy is not free from experimental drawbacks. First, although our COVASP transfer method has been validated as preserving in broad terms the structure of the films in situ (Wang et al., 2007), the transference of the films onto solid supports may cause some artifacts upon accumulation of three‐dimensional structures under the dehydrated transferred layers. Second, our observations are based on the preferential partition of the two fluorescent dyes selected, but that does not mean that the interfacial films comprise only two different types of lipid regions as defined by each probe. Third, the inclusion and, even further, the compression‐driven accumulation of the probes in certain regions of the films likely perturb their organization with respect to the structure of the films in the absence of probes.

## 4 DISCUSSION

To fulfill its biological task, the structural hallmark of PS films is its dynamism to form solid‐like interfacial films at the very end of exhalation. In this process, two events are critical: (1) enrichment of the air‐exposed interfacial monolayer in DPPC to reach very low *γ*_min_ (around 1 mN/m) and (2) exclusion of unsaturated lipids from the interface (Pérez‐Gil, 2008). Furthermore, some lipid/protein complexes are eventually detached from the alveolar interface and recycled by type II pneumocytes and alveolar macrophages (Olmeda et al., 2017). Concurrently, newly synthesized surfactant complexes are secreted and incorporated into the surface film to ensure operative surfactant availability in the alveolar spaces (Olmeda et al., 2017).

Two theories are nowadays under discussion to explain how the interfacial monolayer enriches in DPPC. On the one hand, the squeeze‐out theory argues that lipids are all cooperatively transferred into the alveolar interface, and afterward, unsaturated lipids are preferentially squeezed out during compression (Klenz et al., 2008; Schram et al., 2003; Walters et al., 2000). Thereafter, the interfacial monolayer would be ready to behave similar to a pure DPPC film. On the other hand, there is another trend of thought gaining momentum proposing that DPPC could be selectively transferred into the alveolar air–liquid interface (Possmayer et al., 2023). To explain that, it has been proposed that the so‐called Lγ phase, which is formed under certain circumstances such as low hydration and protein concentration, may provide with a DPPC‐enriched leaflet ready to adsorb into the interface without a need of compositional refinement, from the first breath (Kumar et al., 2018).

Despite seeming contradictory, several experimental evidences support each of these models depending on the materials and the methodology used. Therefore, to unravel the biological mechanism of PS adsorption and primary film formation, the study of pristine surfactant complexes like those isolated from amniotic fluid could represent better the true initial steps in PS physiology.

It has been previously stated that surfactant isolated from porcine lungs is constituted by a mixture of lipid assemblies, with a very minor contribution of the actual pristine surfactant complexes freshly secreted by pneumocytes (Castillo‐Sánchez, Cerrada, et al., 2022). Now that AFS has been shown to present the very same composition than NS in terms of phospholipid classes, ratios of saturated: unsaturated species, and protein amounts but still preserve the features of a freshly secreted surfactant (Castillo‐Sánchez, Roldán, et al., 2022) (summarized in Table 1), we have studied here the structure of interfacial PS films in order to understand how membrane structure in bulk may govern film formation and the structure of air‐exposed films assembled by NS or AFS complexes.

Our results apparently show that interfacial NS films are actually refined during surface cycling in a process equivalent to what we have called the squeeze‐out (Figures 3, 4, 5, 6). After adsorption, NS films seem to be structured as a predominant disordered‐like lipid phase at low Π. It is not until the surface area was compressed to 38 mN/m that the disordered‐like lipid phase (blue arrows in Figure 3) was restrained to defined regions. These label‐enriched regions might then represent local exclusion points, which are formed while the monolayer enriches in DPPC and the film evolves to an ordered‐like phase. However, after surface cycling, the structure of NS films significantly changed since circular‐shaped condensed domains excluding the NBD‐PC dye were observed (black arrows in Figure 4). Therefore, our results illustrate that NS films may certainly undergo a compositional refinement, that is, to get a higher DPPC proportion and reduced unsaturated phospholipid content, during surface cycling, leading to the formation of condensed lipid domains now since relatively low Π values. In this line, and after surface cycling, exclusion points seem to occupy less area percentage, meaning a less need of squeeze‐out because of previous refinement (Figure 5).

Conversely, interfacial AFS films are intrinsically different (Figures 3, 4, and S1). First, we observed that the deposition under comparable conditions of equivalent surfactant mass consistently resulted in higher Π values for AFS than for NS (Figures 1 and 2). A possible reason might be that the adsorption of AFS leads already to higher interfacial DPPC content due to selective adsorption. Moreover, despite the limited spatial resolution in our experiments, we observed that the surface area was already covered by a network of two lipid phases before and after cycling (Figures 3 and 4). Therefore, our fluorescence experiments suggest that AFS films could be structured from the beginning as a material with *L*_o_/*L*_d_ lipid‐phase coexistence after adsorption (Baoukina et al., 2012, 2014; Kim et al., 2013), in contrast to NS, which just comprised a dominant disordered‐like phase, at least under the particular conditions defined by the geometry of our surface balances.

Furthermore, in our fluorescence experiments, we observed circular‐shaped label‐enriched regions at lower Π for AFS films before surface cycling (blue arrows in Figure 3). These regions might then mark the position of upcoming LBPs that remain under the interfacial monolayer until lipid‐free regions are created on expansion, allowing adsorption of new material. This possibility would sustain the adsorption mechanism proposed for LBPs by Haller and collaborators: a self‐regulated process modulated by surface forces so that, once the film is formed, upcoming LBPs cannot unfold and remain somehow connected underneath the interfacial film (Haller et al., 2004).

It is also noteworthy the 2D‐to‐3D conversion mechanism occurring in NS and AFS films at Π values above 45 mN/m (Figure 7). On the one hand, NS films formed heterogeneous, large, and elongated exclusion points immersed in an ordered‐like phase. On the other hand, AFS films are characterized by a homogeneous distribution of ordered‐like regions, immersed in a solid‐like phase, and enclosing bright exclusion points that may represent collapsed structures due to the high Π. We hypothesize that these regions observed in AFS films may maximize the squeeze‐out of fluid lipids, in line with results published by Zhang and collaborators regarding the collapse mechanism of films formed by clinical surfactant preparations (Zhang et al., 2011).

In summary, from the present results, we propose that pulmonary surfactant complexes could be primarily assembled into the LBs in a highly dehydrated and packed form, particularly active to adsorb and transfer into the interface once secreted by the pneumocytes into the alveolar spaces (Haller et al., 2004), as pointed out by the results obtained with AFS. This highly active state, perhaps already containing segregated almost pure DPPC patches, would be responsible for producing the significantly higher surface pressures and interfacial packing observed here compared with that of surfactant complexes isolated from lung lavages. The particularly efficient adsorption of AFS complexes might promote the formation of films with higher interfacial lipid densities (reduced area/molecule) from the mere adsorption, and therefore more condensed states than films formed by NS.

As both NS and AFS present fully comparable compositions in terms of lipids and proteins (Castillo‐Sánchez, Roldán, et al., 2022), we think that the direct comparison of interfacial structural differences performed here is of high relevance. Furthermore, it is worth stressing that both NS and AFS had to be studied from aqueous preparations in order to maintain their intrinsic architecture. However, we do not consider that the differences observed here in terms of adsorption capabilities could be related to the methodological approach of spreading aqueous surfactant solutions into the surface of the Langmuir trough. Both PS materials seem to be so surface‐active that most of them seem to incorporate and remain at the interface upon injection under the different spreading conditions evaluated (Figure 1) (Castillo‐Sánchez, Roldán, et al., 2022).

A limitation has been that the experiments presented in here were performed at 25°C in order to avoid temperature gradients and subphase evaporation in the large surface balances required to prepare the transferred COVASP films compressed to well‐controlled rates and extents. At higher physiological temperatures, the compression‐driven dynamics sustaining phase separation, the three‐dimensional exclusion of the most unstable components, and the achievement of the lowest surface tensions (highest pressures) may differ, at least kinetically. Although technically challenging, future experimentation at physiological temperatures and physiological compression–expansion rates may provide further details into the structural organization of AFS films at the interfacial level. Still, we are confident that similar conclusions regarding the enhanced capabilities of AFS to form highly condensed interfacial surface‐active structures will be reached, in line with previous results obtained under more physiologically comparable setups (Castillo‐Sánchez, Roldán, et al., 2022). Future experiments could also confirm our results by analyzing the structure and behavior of surfactant isolated from other sources, such as porcine amniotic fluid or surfactant purified from lavage of healthy human lungs, that is, obtained from organ donors.

Despite the limitations, we conclude from the results of this study that surfactant films formed by freshly secreted, pristine, and non‐used surfactant such as that represented by AFS could be intrinsically different from all the surfactant films typically formed by NS, clinical surfactants, or model lipid or lipid/protein films. AFS interfacial films could be representative of the true material responsible for a proper mechanical behavior of alveolar spaces under breathing mechanics and of the material that needs to be restored or replicated in order to properly treat patients suffering from lung injury, inflammation, and edema, conditions associated with surfactant inactivation.

## AUTHOR CONTRIBUTIONS

J. C. Castillo‐Sánchez: Investigation, formal analysis, writing—original draft. A. Collada: Investigation, formal analysis, writing—original draft. E. Batllori‐Badia: Resources. A. Galindo: Resources. A. Cruz: Conceptualization, formal analysis, writing—review & editing, supervision. J. Pérez‐Gil: Conceptualization, writing—review & editing, supervision, project administration, funding acquisition.

## FUNDING INFORMATION

This work has been funded by a grant from the Spanish Ministry of Science and Innovation (PID2021‐124932OB‐I00).

## CONFLICT OF INTEREST STATEMENT

Authors declare no conflict of interest.

## ETHICS STATEMENT

The human amniotic fluid used to prepare the material of this work was obtained from full‐termed programmed caesareans of donor mothers upon written informed consent that was presented and approved by the Ethical Committee of Hospital 12 Octubre (CEI 16/205 ‐ 14/07/2016) in Madrid, Spain.

## Supporting information


Figure S1.

## Data Availability

Figure S1, with images taken at different surface pressures from two other different batches of either NS or AFS, is available. Any other data will be made available on request.
