# Supplementary material for: The pristine unused pulmonary surfactant isolated from human amniotic fluid forms highly condensed interfacial films
Source: Physiol Rep. 2025 Jun 19;13(12):e70403. doi: 10.14814/phy2.70403 (PMC12179403; doi:10.14814/phy2.70403)
Supplement: Supplementary file 1 — Figure S1. [file PHY2-13-e70403-s001.zip › PHYSREP-2025-05-381-T-f08-z-.pdf]

SUPPLEMENTARY INFORMATION FOR:

The pristine unused pulmonary surfactant isolated from human amniotic fluid forms highly condensed interfacial films

Castillo-Sánchez JC[1,2§], Ainhoa Collada[1,2§], Batllori-Badia E[3], Galindo A[2,3,4], Cruz A[1,2*], Pérez-Gil J[1,2]*

1 Biochemistry and Molecular Biology Department, Faculty of Biology, Complutense University, Madrid, Spain

2 Research Institute Hospital 12 de Octubre (imas12), Madrid, Spain

3 Department of Public and Maternal-Child Health. Faculty of Medicine. Complutense University of Madrid. 12 de Octubre University Hospital

4 Maternal and Child Health and Development Research Network (RICORS-SAMID network) ref. RD21/0012/0024

§These authors have contributed equally to this work

*Corresponding authors: Jesús Pérez-Gil
Dept. Biochemistry, Faculty of Biology
Complutense University
Jose Antonio Novais 12
28040 Madrid, Spain
Ph: +34 913944994

Antonio Cruz
Dept. Biochemistry, Faculty of Chemistry
Complutense University
28040 Madrid, Spain
Ph: +34 913944156

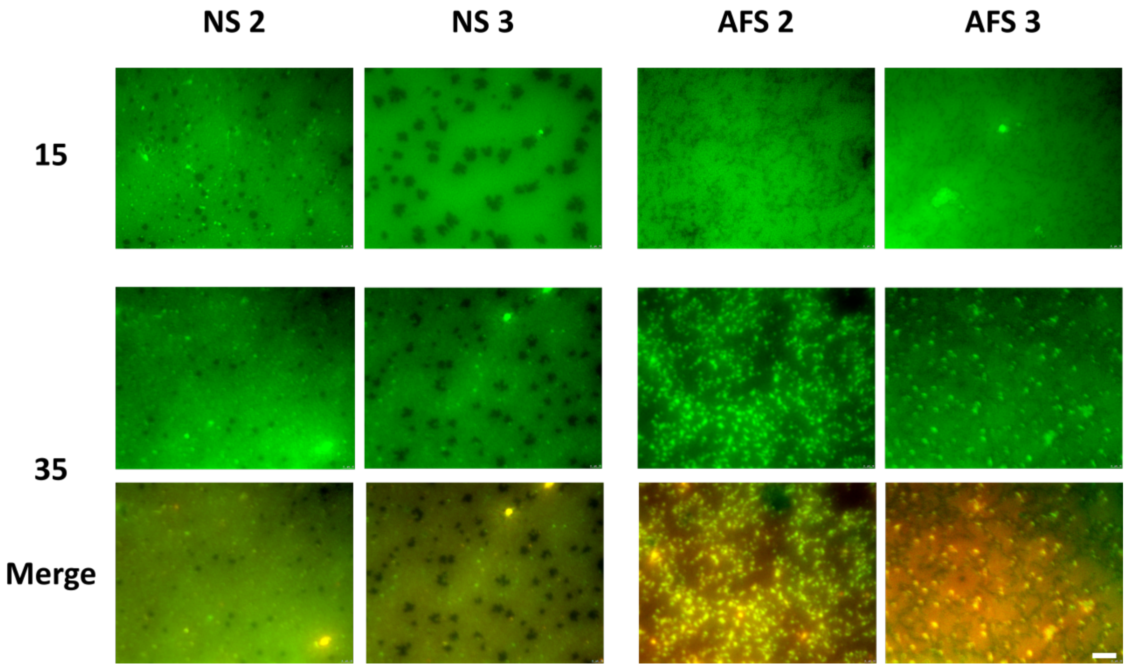

Figure S1. Epifluorescence images were taken at surface pressures of 15 or 35 (in mN/m) from films transferred by the COVASP method for two batches of NS (left) and two batches from AFS (right) different from the ones used to obtain the images of the figures in the main text. For the highest surface pressure, the merge between the fluorescence emission of NBD-PC (green) and DiIc18 (red) is also included to better visualize different lipid phases in the samples. Scale bar means 10 µm.
