# Supplementary figures and images for: The pristine unused pulmonary surfactant isolated from human amniotic fluid forms highly condensed interfacial films
Source: Physiol Rep. 2025 Jun 19;13(12):e70403. doi: 10.14814/phy2.70403 (PMC12179403; doi:10.14814/phy2.70403)

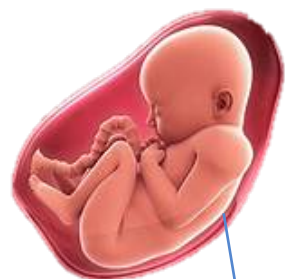

Amniotic fluid  
**AFS**

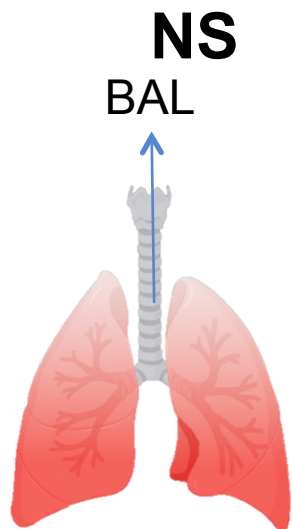

**NS**  
BAL

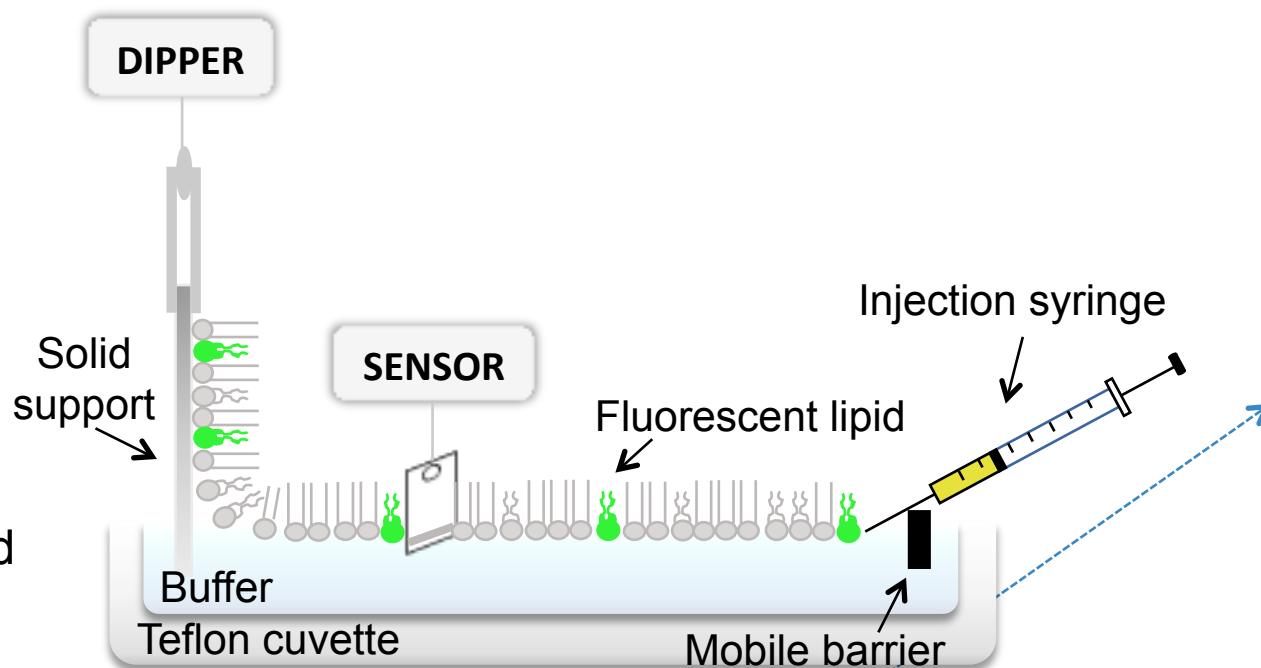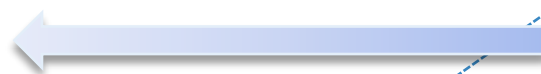

**Compression isotherm**

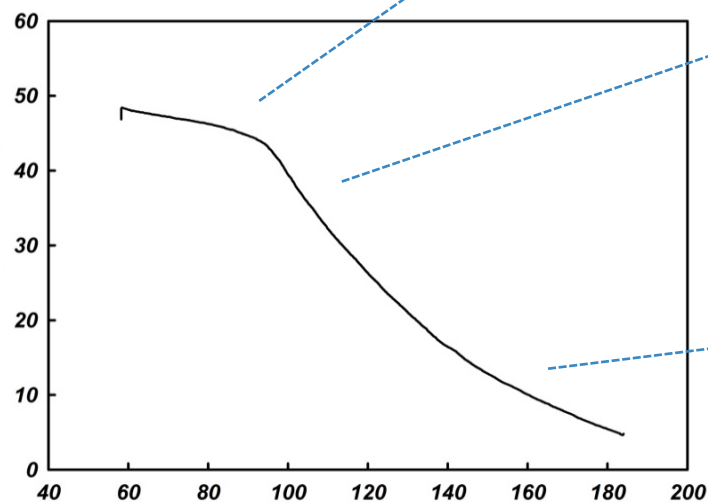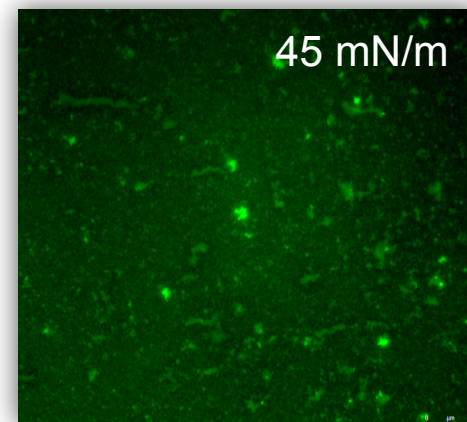

45 mN/m

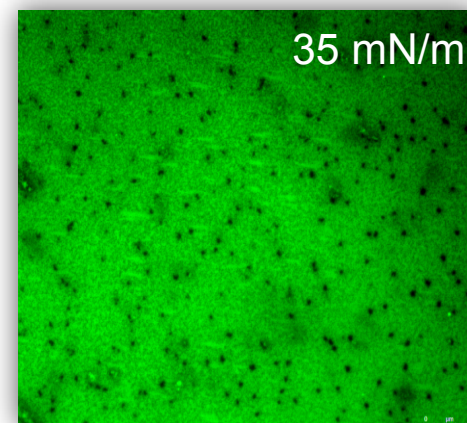

35 mN/m

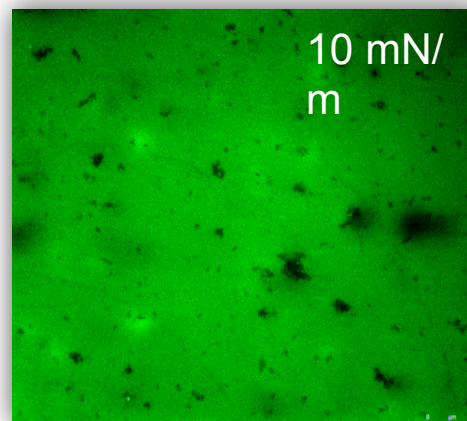

10 mN/  
m

Compression  
driven  
lipid sorting

Supplement: Supplementary file 1 — Figure S1. [file PHY2-13-e70403-s001.zip › PHYSREP-2025-05-381-T-s02.pdf]
